# Protective Allele for Multiple Sclerosis HLA-DRB1*01:01 Provides Kinetic Discrimination of Myelin and Exogenous Antigenic Peptides

**DOI:** 10.3389/fimmu.2019.03088

**Published:** 2020-01-17

**Authors:** Azad Mamedov, Nadezhda Vorobyeva, Ioanna Filimonova, Maria Zakharova, Ivan Kiselev, Vitalina Bashinskaya, Natalia Baulina, Alexey Boyko, Alexander Favorov, Olga Kulakova, Rustam Ziganshin, Ivan Smirnov, Alina Poroshina, Igor Shilovskiy, Musa Khaitov, Yuri Sykulev, Olga Favorova, Valentin Vlassov, Alexander Gabibov, Alexey Belogurov

**Affiliations:** ^1^Shemyakin-Ovchinnikov Institute of Bioorganic Chemistry, Russian Academy of Sciences, Moscow, Russia; ^2^Institute of Gene Biology, Russian Academy of Sciences, Moscow, Russia; ^3^Pirogov Russian National Research Medical University, Moscow, Russia; ^4^Neuroimmunological Department of the Federal Center of Cerebrovascular Diseases and Stroke, Moscow, Russia; ^5^Sidney Kimmel Comprehensive Cancer Center, The Johns Hopkins University, Baltimore, MD, United States; ^6^Vavilov Institute of General Genetics, Russian Academy of Sciences, Moscow, Russia; ^7^Institute of Fundamental Medicine and Biology, Kazan (Volga) Federal University, Kazan, Russia; ^8^National Research Center Institute of Immunology FMBA of Russia, Moscow, Russia; ^9^Department of Microbiology, Thomas Jefferson University, Philadelphia, PA, United States; ^10^Institute of Chemical Biology and Fundamental Medicine, Siberian Branch of Russian Academy of Sciences, Novosibirsk, Russia; ^11^Department of Fundamental Medicine, Lomonosov Moscow State University, Moscow, Russia

**Keywords:** myelin basic protein, multiple sclerosis, human leukocyte antigen, protective allele, epitope library, hemagglutinin, genetic predisposition to disease

## Abstract

Risk of the development of multiple sclerosis (MS) is known to be increased in individuals bearing distinct class II human leukocyte antigen (HLA) variants, whereas some of them may have a protective effect. Here we analyzed distribution of a highly polymorphous HLA-*DRB1* locus in more than one thousand relapsing-remitting MS patients and healthy individuals of Russian ethnicity. Carriage of HLA-DRB1*15 and HLA-DRB1*03 alleles was associated with MS risk, whereas carriage of HLA-DRB1*01 and HLA-DRB1*11 was found to be protective. Analysis of genotypes revealed the compensatory effect of risk and resistance alleles *in trans*. We have identified previously unknown MBP_153−161_ peptide located at the C-terminus of MBP protein and MBP_90−98_ peptide that bound to recombinant HLA-DRB1*01:01 protein with affinity comparable to that of classical antigenic peptide 306-318 from the hemagglutinin (HA) of the influenza virus demonstrating the ability of HLA-DRB1*01:01 to present newly identified MBP_153−161_ and MBP_90−98_ peptides. Measurements of kinetic parameters of MBP and HA peptides binding to HLA-DRB1*01:01 catalyzed by HLA-DM revealed a significantly lower rate of CLIP exchange for MBP_153−161_ and MBP_90−98_ peptides as opposed to HA peptide. Analysis of the binding of chimeric MBP-HA peptides demonstrated that the observed difference between MBP_153−161_, MBP_90−98_, and HA peptide epitopes is caused by the lack of anchor residues in the C-terminal part of the MBP peptides resulting in a moderate occupation of P6/7 and P9 pockets of HLA-DRB1*01:01 by MBP_153−161_ and MBP_90−98_ peptides in contrast to HA_308−316_ peptide. This leads to the P1 and P4 docking failure and rapid peptide dissociation and release of empty HLA-DM–HLA-DR complex. We would like to propose that protective properties of the HLA-DRB1*01 allele could be directly linked to the ability of HLA-DRB1*01:01 to kinetically discriminate between antigenic exogenous peptides and endogenous MBP derived peptides.

## Introduction

Human leukocyte antigen (HLA) genes encode proteins that are capable to bind and present antigenic peptides and, therefore, play a critical role in the immune responses against pathogens as well as those resulting in autoimmunity (1–4). Binding of antigenic peptides to HLA class II molecules produces binary peptide-HLA ligands displayed on the cell surface for recognition by T-cell receptors (5). Initially, nascent HLA proteins are protected by the invariant chain (6). In the endosome compartment, the invariant chain is partially degraded leaving HLA class II-associated Ii peptide (CLIP) in the binding groove (7, 8). Peptide antigens that are processed in the endosomes could then exchange with CLIP bound to the HLA molecules, a process that is facilitated by HLA-DM protein (9, 10). Finally, the peptide-HLA II complex is translocated to the surface of the antigen presenting cell (APC) for survey by T cells. While mechanisms of peptide presentation by HLA class II proteins is well-understood (11, 12), how generation and presentation of self-peptide-HLA class II ligands results in the development of autoimmune reactions is still unclear and remains a subject of a great interest. Indeed, identification of self-peptide-HLA class II ligands that are linked to autoimmune reactions promises to provide a clue for understanding of the pathogenesis of autoimmune disorders (13–15).

Multiple sclerosis (MS), a chronic autoimmune disease of the central nervous system (CNS), which is characterized by inflammation, demyelination, and neurodegeneration (16). The nature of genetic susceptibility to MS is complex and depends on the interplay between multiple genetic, epigenetic, and environmental factors (17). Since the early 2000s, genome-wide association studies have been exploited as a powerful tool for investigating the genetic basis of MS and have revealed more than 200 disease-associated loci; however, genes within HLA region are thought to exert a major genetic contribution to MS risk (18). Particular alleles of the highly polymorphous HLA class II *DRB1* gene appear to be the strongest genetic determinant for MS and may influence both predisposition and resistance to the disease (19). Genetic heterogeneity in MS patients were observed in different populations. For instance, the HLA-DRB1*15:01 allele and its associated haplotype (DQB1*06:02, DQA1*01:02, DRB1*15:0, DRB5*01:01) have been known as a near-universal MS risk factor since the 1970s. Analysis of the HLA associations in Northern European MS populations uncovered many other HLA-*DRB1* alleles (DRB1*03, *01, *10, *11, *14, *08) that were either positively or negatively associated with the disease (20). The distinct autoantigenic peptides presented by predisposing alleles have been identified. For instance, DRB1*15:01 binds peptide from myelin basic protein (MBP), i.e., MBP_85−99_ peptide (14), while DRB5*01:01 presents MBP_86−105_ peptide (13) and DRB1*04:01 can display MBP_111−129_ peptide (21). While these findings define disease-associated peptide-HLA ligands recognizable by T-cells (21–23), the mechanism providing resistance to MS by protective HLA alleles is not known. Here, on a representative cohort of ethnically Russian MS patients and healthy individuals, we show that group of alleles HLA-DRB1*01 is associated with resistance to MS. We have identified a novel MBP-derived peptide ligand presented by a particular HLA-DRB1*01:01 protein and have shown, that this HLA class II protein can kinetically discriminate between the MBP and virus peptides suggesting a mechanism responsible for resistance to MS of individuals that carry HLA-DRB1*01 alleles.

## Materials and Methods

### Patients and Controls

Five hundred and sixty five unrelated relapsing-remitting MS (hereinafter referred to as “MS”) patients from Moscow Multiple Sclerosis Center diagnosed according to the McDonald Criteria (24) and self-reported as Russians were selected for the study. Four hundred and seventy-one healthy individuals without neurological disorders and familial history of MS were included in the control group; they were also self-reported as Russians. All MS patients and healthy individuals lived in the Moscow region. Demographic and clinical data for all participants are presented in Table 1. No significant differences in demographic characteristics (age and sex ratio) were observed between two groups. This study was carried out in accordance with the recommendations of local ethics committee of the Moscow Multiple Sclerosis Center. All subjects gave written informed consent in accordance with the Declaration of Helsinki. The protocol #13 from 15 September 2014 was approved by the local ethics committee of the Moscow Multiple Sclerosis Center.

**Table 1:** Demographic and clinical data for relapsing-remitting multiple sclerosis patients and healthy individuals (all Russians).

	**RRMS**, ***n* = 565**	**Healthy individuals**, ***n* = 471**
Sex ratio (female/male)	2.3:1 (395/170)	1.9:1 (310/161)
Age (years), mean ± SD	38.8 ± 10.6	44.2 ± 16.4
Individuals with familial history of MS (%)	27 (4.8%)	0
Age at onset (years), mean ± SD	27.4 ± 9.2	–
Disease duration (years), mean ± SD	11.4 ± 7.4	–
EDSS, mean ± SD	2.8 ± 1.2	–
MSSS, mean ± SD	3.4 ± 1.9	–

*EDSS, Expanded Disability Status Scale; MSSS, Multiple Sclerosis Severity Score; SD, Standard Deviation; RRMS, Relapsing-Remitting Multiple Sclerosis*.

### Genotyping and Statistical Analysis

Genomic DNA was extracted from the peripheral blood samples with QIAamp DNA Blood Midi Kits (QIAGEN). Low-resolution (two-digital) genotyping of the HLA-*DRB1* locus was performed using HLA-DRB1 real-time PCR Genotyping Kit (DNA-Technology). HLA-*DRB1* alleles and genotypes associated with risk of MS were identified using the APSampler software [http://apsampler.sourceforge.net/] and validated with Fisher's exact test included in the software. Association was considered nominally significant; if uncorrected *p*-values (*p*_*f*_) for identified alleles/genotypes were <0.05 and 95% confidence interval (CI) for odds ratio (OR) did not cross 1. A standard permutation test with 100 APSampler runs was performed for each finding as a multiple hypothesis testing correction. The significance threshold was set at *p*_*perm*_ <0.05.

### Preparation of Human Dendritic Cells and Identification of Bound Peptides

The fraction of mononuclear cells (PBMC), containing dendritic cells (DC) progenitors, were isolated from human blood according standard protocol (25). 20–50 ml of blood from each donor was diluted 3 times with PBS-EDTA (PBS with 2 mM EDTA), carefully underlayered with 1/4 of volume with Ficoll solution (1.077 g/cm^3^, Paneco) and centrifuged at 750 g for 30 min at room temperature. The dense band of PBMCs was removed carefully, placed into 50 ml tube, diluted 3 times with PBS-EDTA, centrifuged at 200 g for 10 min at 4°C and the cell pellet was once washed with PBS-EDTA. Then it was solved in RPMI advanced medium with 10% bovine fetal serum, glutamax and antibiotic-antimicotic (ThermoFisher Scientific), seeded in 25 cm^2^ cultural flasks in 6*10^6^ cell/ml concentration. After 2 h the unbound cells were removed and media was changed to fresh portion with DC growth factors—IL4 (100 ng/ml) and GM-CSF (50 ng/ml) (StemCells) and then cultivated for 6 days with a change of 1/2 of media volume each second day as described Markov et al. (26). After 6 days full media volume was changed to fresh portion with Bacterial LipoPolysaccharide (10 mkg/ml) and cultivated for 24 h for DC maturation. After 7 days DC were unbound by cell scrapper, lysed in PBS with 0.25% of sodium deoxycholate in presence of complete EDTA-free inhibitors (Roche), PMSF, Pepstatin, EDTA for 1 h at 4°C with following centrifugation at 16,000 g for 20 min. Then cell lysates were applied onto size-exclusion chromatography column Superdex75 (GE Healthcare). Presence of MHC II molecules in several high-molecular fractions, corresponding to MHCII tetramers, were verified by ELISA, where MHCII molecules were defined by binding with pre-immobilized mouse L243 antibody (anti-MHCII) and following successive interaction with rabbit anti-MHCII polyclonal serum and with anti-rabbit anti-whole molecule antibody-HRP (Sigma). These fractions were lyophilized.

### LC-MS/MS and DATA Analysis

Isolated peptides were desalted using SDB-RPS StageTips as it was described earlier (27). LC-MS/MS analysis was performed using the Q Exactive HF benchtop Orbitrap mass spectrometer (Thermo Fisher Scientific) which was coupled to the Ultimate 3000 Nano LC System (Thermo Fisher Scientific) via a nanoelectrospray source (Thermo Fisher Scientific). The HPLC system was configured in a trap-elute mode. Peptide solution were loaded on an Acclaim PepMap 100 (100 μm × 2 cm) trap column and separated on an Acclaim PepMap 100 (75 μm × 50 cm) column (both from Thermo Fisher Scientific). Correlation of MS/MS spectra with peptide sequences was made using PEAKS Studio 8.0 build 20160908 software (28). Peptide lists generated by the PEAKS Studio were searched against the *Homo sapiens* Uniprot FASTA database (154257 species, version July 2016) and with methionine oxidations and asparagine/glutamine deamidations as variable modifications. The false discovery rate (FDR) for peptide-spectrum matches was set to 0.01 and was determined by searching a reverse database. Peptide identification was performed with an allowed initial precursor mass deviation up to 10 ppm and an allowed fragment mass deviation of 0.05 Da.

### MHC Expression and Purification

The genetic constructions for recombinant HLA-DR (HLA-DRB1*01:01 and HLA-DRB1*15:01) α and β (with or without CLIP) chains expression were created based on pMT-V5/His and pRmHa vectors, respectively. All HLA-DRs carried parts of leucine zipper from c-jun and c-fos transcription factors as previously described (29). CLIP (PVSKMRMATPLLMQA) was covalently attached with the linker with a thrombin site at the N-terminus of β chain. The individual stable lines of *Drosophila melanogaster* S2 cells, carrying both genes of appropriate α and β (with or without CLIP) HLA-DR chains and separate plasmid pCoBlast (Invitrogen) with blasticidin resistance, were obtained. The HLA-DRB1 proteins with and without CLIP were expressed in SF900 III Media (Gibco) during 3–7 days after induction with 1 mM Cu^2+^ at 28°C with shaking. Then the cell culture concentrate was applied to affinity anti-MHC II (L243) resin in PBS, followed by elution with 50 mM glycine buffer (pH 11.5) and rapid neutralization of eluate by 2 M Tris-HCl (pH 8.0) (29). For the next step, impurities were removed with MonoQ column (GE Healthcare) in 0–1 M NaCl gradient. Constructions for recombinant HLA-DM α and β chain expression in eukaryotic suspension cells, HEK 293F, were previously created based on pFUSE vector encoding constant fragments of human immunoglobulin heavy chain (Fc) (29). The appropriate constructions were used for transit transfection of HEK293F cells with a following expression of HLA-DM, performed in serum-free FreeStyle medium (Gibco) until the percentage of living cells was lower than 60% (typically 5–7 days). Then the concentrated culture medium was loaded into a Protein G affinity column (GE Healthcare), followed by elution with 50 mM glycine buffer (pH 2.5) and rapid neutralization of eluate by 2 M Tris-HCl (pH 8.0). The second purification step comprised the use of an ion-exchange MonoQ column (GE Healthcare), following the same procedure as described above. Proteins were concentrated, transferred to 20 mM Tris-HCl (pH 8.0), 150 mM NaCl buffer and stored at 4°C.

### Thioredoxin-Fused Peptides Expression and Purification

Thioredoxin-fused peptides were constructed and produced earlier as parts of an MBP epitope library (30). Twelve successive overlapping short fragments of MBP (25–30 aa) were placed on the C-terminus of bacterial thioredoxin via flexible linker (SGGGG)_3_S, carrying an His-tag for purification. The substrate construct, carrying only thioredoxin with the linker (TRX), was used as a control. New thioredoxin-fused peptides were created in this work, using MBP epitope library as a template. The genetic constructs, encoding HA, pp65, CLIP, MBP with point mutations and chimeric peptides, were obtained by insertion using overlapping PCR. All thioredoxin-fused substrates were produced in *Escherichia coli* BL21 (DE3) strain in soluble form, purified with Ni-NTA (Qiagen) and MonoS (GE Healthcare) columns (30). Thioredoxin-fused peptides were chemically biotinylated with EZ-Link Sulfo-NHS-LC-biotin (Thermo Fisher Scientific) in molar ratio 1:20 for 30 min at 25°C. Proteins were concentrated in PBS and stored at −20°C.

### Peptide Binding Experiments and Dissociation Constant Determination

Both biotinylated synthetic and thioredoxin-fused MBP peptides (750 nM, which is much higher than measured *K*_D_ to study maximal binding) from the MBP library and with point mutations, were incubated overnight at 37°C in PBS in 50 μl with HLA-DR (HLA-DRB1*01:01 or HLA-DRB1*15:01) (150 nM). DR/peptide complexes were then captured on the ELISA plate with immobilized L243 mAb (5 μg/ml in PBS) and blocked with 1% milk in PBS. HLA-DR-bound biotinylated peptide was quantitated with streptavidin-peroxidase (50 μl in PBST), using 3,3′,5,5′-tetramethylbenzidine (50 μl) as a substrate and stopping with 10% phosphoric acid (50 μl). ELISA was performed using standard protocol (https://www.abcam.com/protocols/sandwich-elisa-protocol).

In a competition assay, biotinylated thioredoxin-fused HA and pp65 peptides (150 nM) were incubated with HLA-DR in the presence of HA, pp65, MBP or chimeric peptides (1,000, 500, 250, 125, 62.5, 31.2, 15.6, and 7.8 nM) for 18 h. Whole procedure was the same as described above.

For *K*_D_ determination, biotinylated synthetic and thioredoxin-fused peptides were incubated in the same conditions as described above with HLA-DR (5 nM) at the following concentrations: 10,000, 3,000, 1,000, 300, 100, 30, 10, 3, and 0.1 nM. DR/peptide complexes were quantitated using Europium-labeled streptavidin and enhancement solution. DELFIA was performed according to standard protocol (http://www.perkinelmer.com/lab-products-and-services/application-support-knowledgebase/delfia/delfia-immunoassays.html). All experiments were carried out in triplicates.

### Kinetic Measurement of Peptide Loading on HLA-DR

A real-time ELISA assay was used to assess kinetics of peptide exchange on HLA-DR, catalyzed by HLA-DM. The linker connecting the CLIP peptide to the N-terminus of the HLA-DR β chain was cut with thrombin (1 h, 20 U/mg, at room temperature). DR-CLIP complexes (150 nM) were incubated with or without HLA-DM (150 nM, which was estimated as minimal concentration for maximal rate, see Figure S1) in the presence of biotinylated either synthetic or thioredoxin-fused HA, pp65, MBP, and chimeric peptides (150 nM, which was experimentally estimated as minimal concentration required for reliable detection), for 7, 5, 3, 1, and 0 h. Each time point was mixed separately starting from the end of incubation time (7 h) every 2 h, afterwards all time points were loaded into the plate.

In a competition assay, kinetic experiments were carried out as described above. Kinetics were measured in the presence of increasing concentrations (0, 30, 100, 300, and 1,000 nM) of thioredoxin-fused HA, pp65, MBP, or chimeric peptides at timepoints 8, 6, 4, 2, and 0 h. Thioredoxin without any peptide was used as a control. ELISA was performed as done previously. All experiments were carried out in triplicates.

## Results

### Genetic Association of HLA-DRB1 Gene Variants With MS

**Figure 1:**
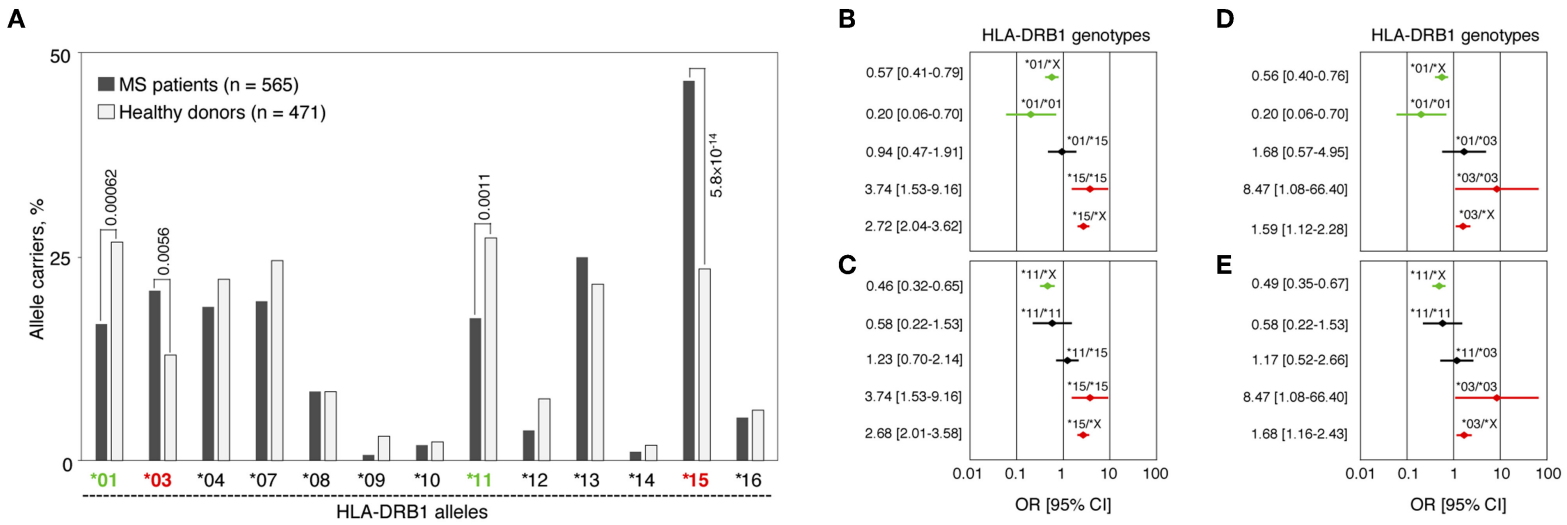
Genetic association of HLA-DRB1 gene variants with MS. **(A)** Carriage frequencies of HLA-*DRB1* alleles in MS patients and healthy controls. *p*_*perm*_ values for alleles DRB1*01, *03, *11 and *15, significantly associated with MS after correction for multiple comparisons, are presented. **(B–E)** Forest plots demonstrate odds ratios (ORs) and their 95% confidential intervals (95%CI) for the HLA-*DRB1* genotypes that include identified MS predisposing and protective alleles as homozygotes and in heterozygous combinations with each other or with the rest of the HLA-*DRB1* alleles. **(B)** HLA-DRB1*01 and *15 alleles; **(C)** HLA-DRB1*11 and *15 alleles; **(D)** HLA-DRB1*01 and *03 alleles; **(E)** HLA-DRB1*11 and *03 alleles. For each plot **(B–E)**, a set of all the *DRB1* alleles excluding the alleles of interest is denoted by the letter “X”; OR [95%CI] values are shown in red for positive association with MS genotypes, in green for negatively associated genotypes and in black for non-significant genotypes.

We analyzed HLA-*DRB1* allelic distribution in 565 MS patients and 471 healthy individuals (all of Russian ethnicity) using low-resolution (two-digital) genotyping. Significant differences in carriage frequencies of several HLA*-DRB1* groups of alleles (hereinafter “alleles”) were observed (Figure 1A and Table S1). Alleles HLA-DRB1*03 and *15 were strongly associated with high MS risk [*p*_*perm*_ = 0.0056, OR = 1.77 (CI: 1.27–2.49) and *p*_*perm*_ = 5.8 × 10^−14^, OR = 2.84 (CI: 2.17–3.72), respectively]. HLA-DRB1*01, *09, *11, and *12 alleles were significantly enriched in the control group. However, only alleles HLA-DRB1*01 and *11 were determined to be protective after correction for multiple comparisons [*p*_*perm*_ = 0.00062, OR = 0.55 (CI: 0.41–0.74) and *p*_*perm*_ = 0.0011, OR = 0.56 (CI: 0.42–0.76), respectively], while others simply remained nominally significant.

We further analyzed frequencies of heterozygous genotypes including one risk allele (HLA-DRB1*03 or *15) and one resistance allele (HLA-DRB1*01 or *11), i.e., *01/*15, *11/*15, *01/*03, and *11/*03 in MS patients and healthy individuals. Neither of those genotypes were associated with MS: 95% CI for OR crossing 1 (Figures 1B–E), while *p*_*f*_-values lay within the interval between 0.25 and 0.51 (not shown). These data suggest the compensatory effect of any identified protective and predisposal alleles *in trans*. Moreover, dose allele effect was observed when homozygotes *01/*01, *03/*03, and *15/*15 were compared with heterozygotes containing HLA-DRB1*01, *03, or *15 alleles in combination with alleles not associated with MS (denoted as “X” on Figures 1B–E). For HLA-DRB1*11 allele (Figures 1C,E), the dose effect was not observed.

Genetic constructs bearing HLA-DRB1*01:01 and *15:01 alleles were used for further analyses since these alleles are the most frequent among corresponding groups of alleles.

### HLA-DRB1*01:01 Binds Encephalitogenic and C-Terminal Peptides of the Myelin Basic Protein

**Figure 2:**
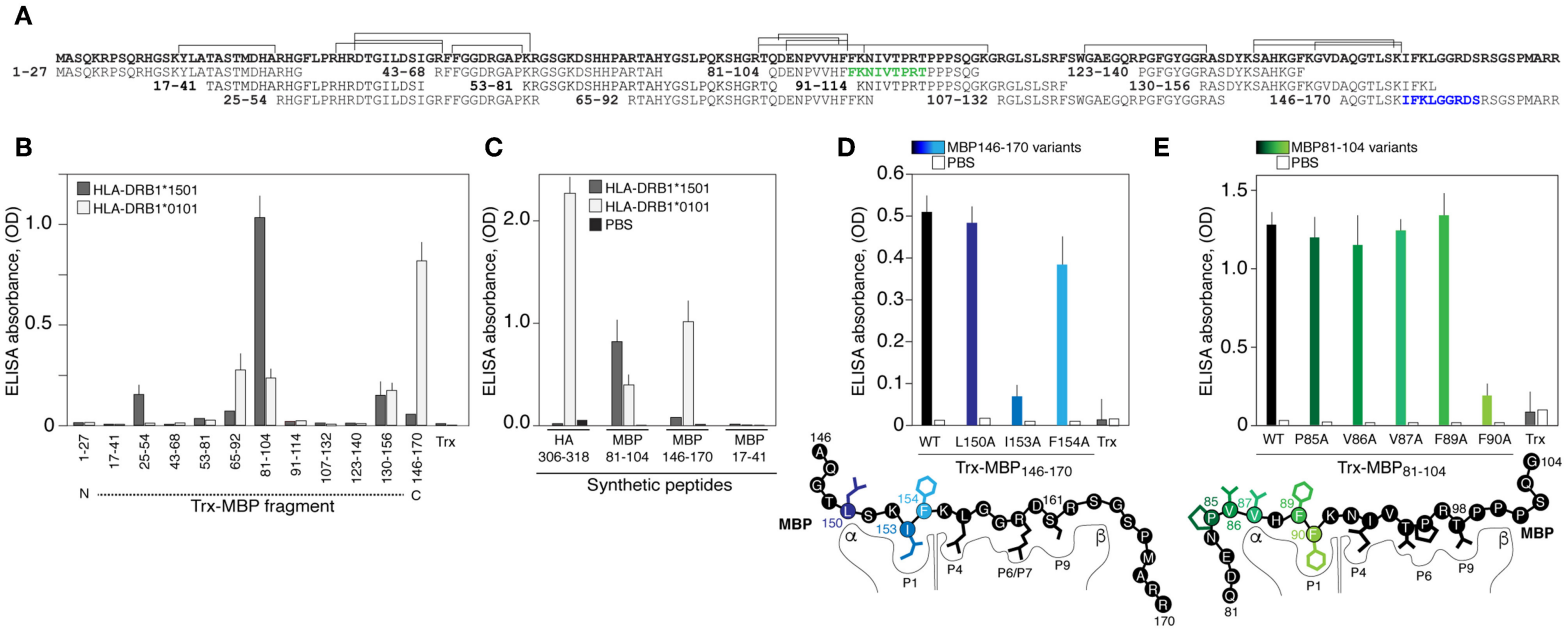
The HLA-DRB1*01:01 recognizes encephalitogenic and C-terminal parts of the myelin basic protein. **(A)** Amino acid sequence of the MBP. Overlapping peptides of the epitope library are indicated. Two epitopes of the HLA-DRB1*01:01 are marked by green and blue. Brackets correspond to the sequences of peptides associated with HLA exposed on the dendritic cells isolated from normal individual with heterozygous genotype HLA-DRB1*01:01/*15:01 according to LC-MS/MS analysis. **(B)** Analysis of binding of HLA-DRB1*15:01 (gray bars) and HLA-DRB1*01:01 (white bars) (150 nM) with the MBP epitope library (750 nM). Trx denotes thioredoxin. Standard deviation is indicated. **(C)** Recognition of the chemically synthesized peptides representing MBP fragments and HA_306−318_ peptide (750 nM) by the HLA-DRB1*15:01 (gray bars) and HLA-DRB1*01:01 (white bars) (150 nM). Black bars represent background signal (PBS). Standard deviation is indicated. **(D,E)** Binding of the thioredoxin-fused peptides and its variants (750 nM) with alanine point substitutions representing MBP_146−170_ **(D)** and MBP_81−104_ **(E)** with HLA-DRB1*01:01 (150 nM). Open bars represent background signal. Standard deviation is indicated. Point mutations are indicated by different overtones.

We further determined if recombinant HLA-DRB1*01:01, encoded by the most common allele among HLA-DRB1*01 groups [http://allelefrequencies.net/hla6006a.asp], may bind MBP-derived peptides utilizing a previously created MBP epitope library (30) (Figure 2A). HLA-DRB1*01:01 specifically bound MBP_146−170_ and less efficiently bound three other peptides MBP_130−156_, MBP_81−104_, and MBP_65−92_ (Figure 2B). As anticipated, HLA-DRB1*15:01 specifically recognized encephalitogenic peptide MBP_81−104_ (Figure 2B). Two more fragments, namely MBP_25−54_ and MBP_130−156_, were bound by HLA-DRB1*15:01; however, with significantly diminished efficiency. Obtained data were verified utilizing chemically synthesized biotinylated peptides (Figure 2C). The HLA-DRB1*01:01 recognized its classical ligand–immunodominant peptide 306-318 of the hemagglutinin (HA) of the influenza virus (31), and both myelin peptides, MBP_81−104_ and MBP_146−170_. The HLA-DRB1*15:01 bound only MBP_81−104_, and neither HLA-DRB1*01:01 nor HLA-DRB1*15:01 bound irrelevant MBP_17−41_ peptide. LC-MS/MS analysis of peptides associated with HLA exposed on the dendritic cells isolated from normal individual with heterozygous genotype HLA-DRB1*01:01/*15:01 revealed three main MBP regions, which corresponded to MBP_81−104_, MBP_25−54_, and MBP_130−156_, that were shown to be bound by HLA-DRB1*15:01. At the same time, we failed to detect any peptides related to MBP_146−170_, bound by HLA-DRB1*01:01 (Figure 2A). In line with these findings peptide MBP_146−170_ did not activate proliferation of CD4-positive T cells according to the CFSE assay (Figure S2).

### Myelin Peptides Recognized by HLA-DRB1*01:01 Contained Polar Residues in P6/P7 and P9

Next, it was important to determine binding epitopes recognized by HLA-DRB1*01:01, which are present in MBP_81−104_ and MBP_146−170_. Alanine scanning (substitutions of hydrophobic and aromatic residues) of the thioredoxin-fused MBP_146−170_ peptide (Figure 2D) revealed that the P1 pocket in the HLA-DRB1*01:01 carrying MBP_146−170_ is occupied by isoleucine at position 153 (human MBP_1−170_ nomenclature). Polar arginine and serine at positions 159 and 161 represent the P6/P7 and P9 residues, respectively. Alanine scanning of the thioredoxin-fused MBP_81−104_ peptide (Figure 2E) resulted in the determination of the phenylalanine at position 90 as a hydrophobic P1 anchor; in turn, this suggested that P6/P7 and P9 pockets in the HLA-DRB1*01:01 bound with MBP_81−104_ are occupied by threonine, proline, and threonine at positions 95, 96, and 98, respectively.

### Loading of the HLA-DRB1*01:01 by Viral and Myelin Peptides Is Thermodynamically but Not Kinetically Equal

**Figure 3:**
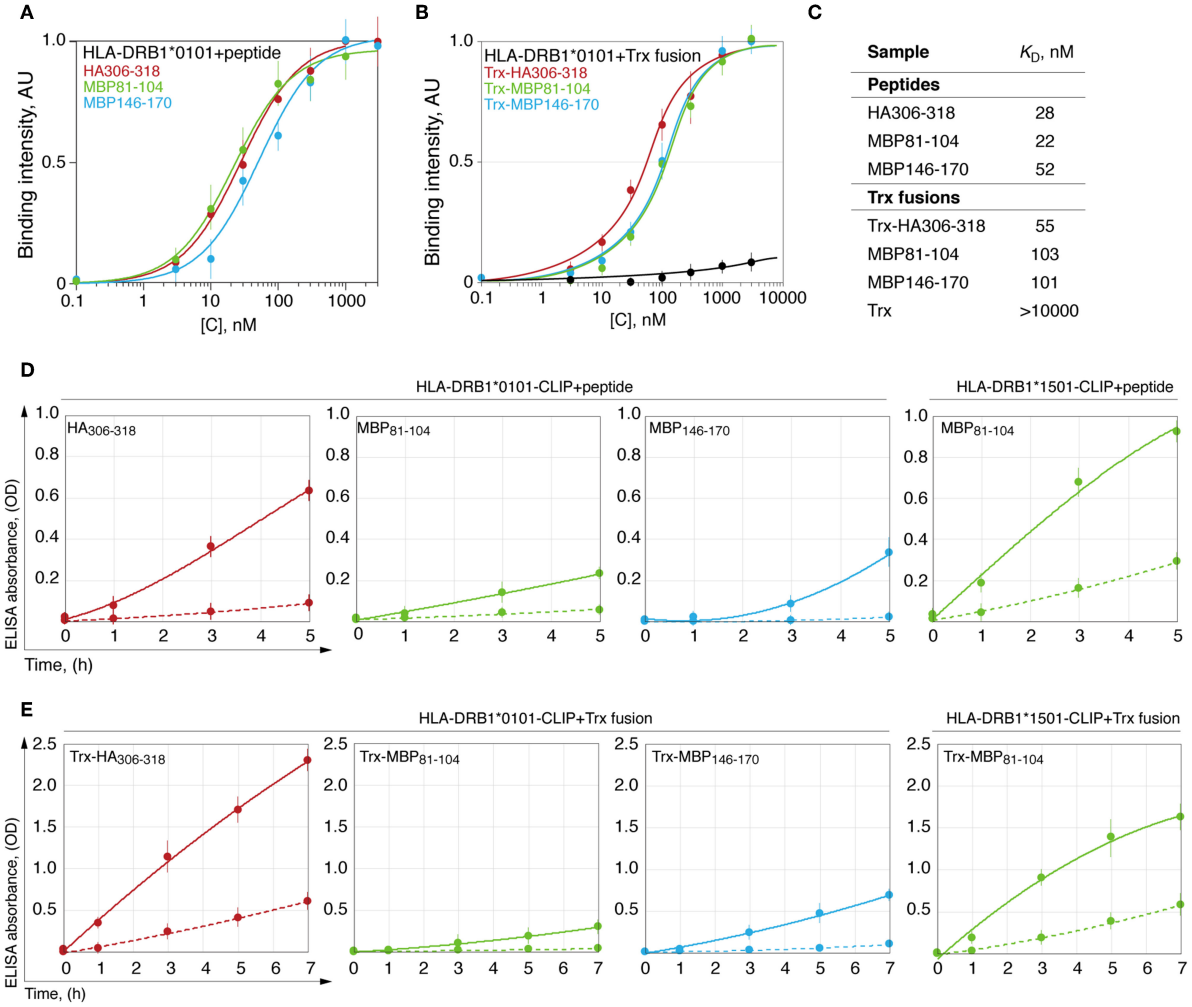
HLA-DRB1*01:01 kinetically discriminates peptides with similar thermodynamic affinities. Binding of chemically synthesized peptides HA_306−318_ (red curve), MBP_81−104_ (green curve) and MBP_146−170_ (blue curve) **(A)** and their thioredoxin-fused analogous **(B)** in different concentrations (0.1 nM−3 μM) with HLA-DRB1*01:01 (5 nM) measured by DELFIA with time-resolved fluorescence detection. AU denotes arbitrary units and Trx represents thioredoxin. Dissociation constants are summarized in **(C)**. Kinetics of binding of chemically synthesized peptides **(D)** and their recombinant analogous as thioredoxin fusions **(E)** containing HA_306−318_ (red curve), MBP_81−104_ (green curve), and MBP_146−170_ (blue curve) (150 nM), to preloaded with CLIP HLA-DRB1*01:01 and HLA-DRB1*15:01 (150 nM) with (solid curves) or without (dashed curves) HLA-DM (150 nM).

Dissociation constant (*K*_D_) of the HLA-peptide complex may characterize strength, which is required to remove a fully docked peptide from the HLA binding groove. To determine this, we measured *K*_D_ for the complexes of the HLA-DRB1*01:01 with myelin peptides MBP_81−104_, MBP_146−170_, and viral HA_306−318_. For this purpose, we utilized both chemically synthesized peptides (Figure 3A) and thioredoxin-fused recombinant epitopes (Figure 3B). Calculated values of the *K*_D_ revealed that HLA-DRB1*01:01 binds all epitopes with approximately similar efficacy (Figure 3C).

Removing the CLIP and docking of the antigenic peptides on the HLA class II is a dynamic process that is catalyzed by the HLA-DM (32, 33). During this process, despite similar affinity, the HA peptide exchanged CLIP loaded onto HLA-DRB1*01:01 significantly more rapidly in comparison with MBP peptides 81-104 and 146-170, whereas HLA-DRB1*15:01 bound MBP_81−104_ to a similar rate of the HLA-DRB1*01:01–HA interaction (Figure 3D). Study of loading of these peptides as a part of thioredoxin fusion proteins on HLA resulted in identical results (Figure 3E).

### C-Terminal P6/P7 and P9 Residues in Viral and Self-Peptides Make Their Kinetic Discrimination by HLA-DRB1*01:01 Possible

**Figure 4:**
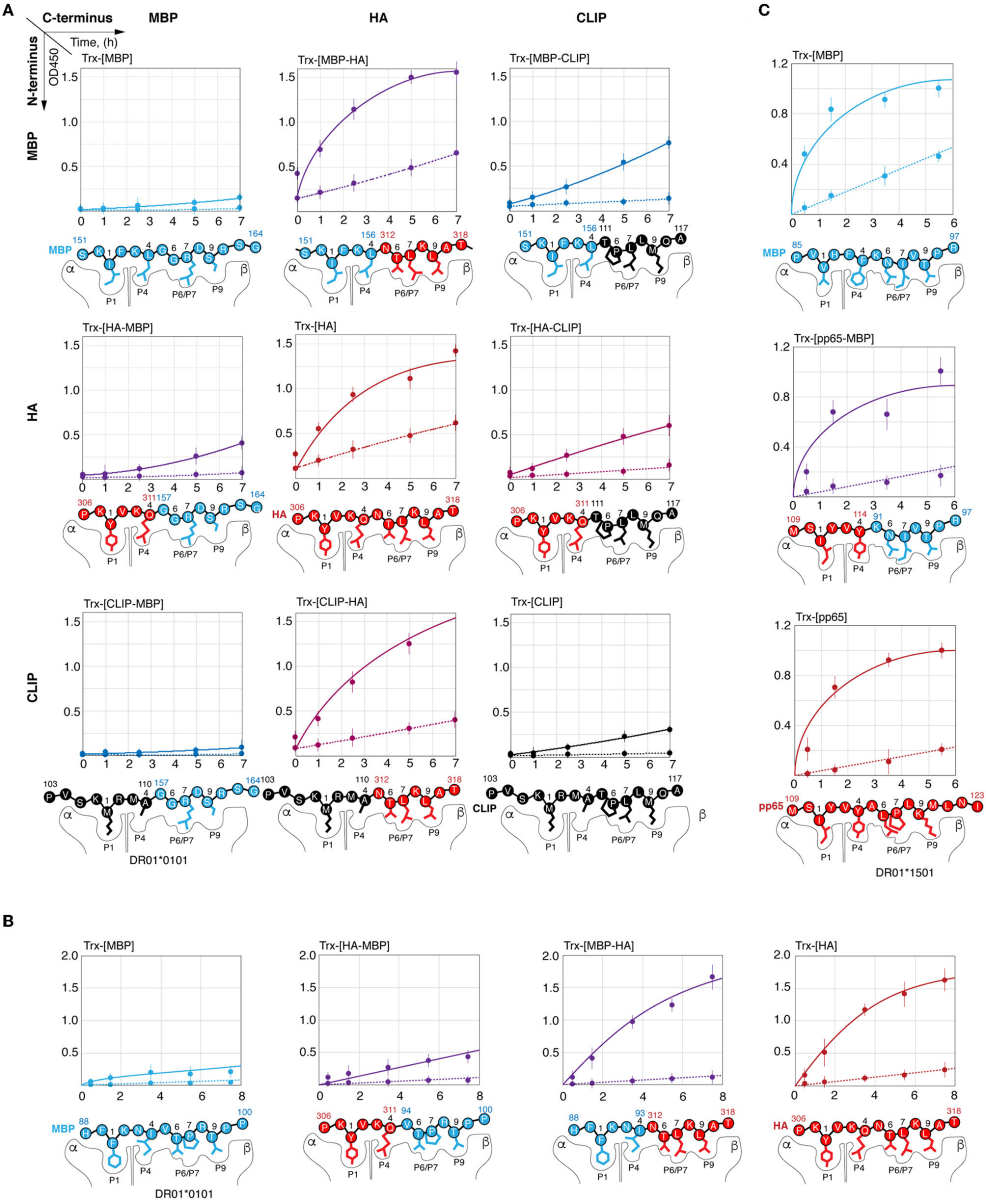
Kinetic discrimination of myelin and viral peptides by HLA-DRB1*01:01 is caused by their C-terminal residues. Kinetics of binding of thioredoxin fusions (150 nM) containing chimeric peptides assembled from N- and C-terminal parts of HA_306−318_ (red), MBP_151−164_ (blue), and CLIP (black) with HLA-DRB1*01:01 (150 nM) **(A)**; of HA_306−318_ (red) and MBP_88−100_ (blue) with HLA-DRB1*01:01 (150 nM) **(B)**; of pp65_109−123_ (red) and MBP_85−97_ (blue) with HLA-DRB1*15:01 (150 nM) **(C)** in the presence (solid lines) or absence (dashed lines) of HLA-DM (150 nM) measured by ELISA. Anchor residues are indicated.

To determine the reason for the slow rate of C-terminal myelin peptide loading on HLA-DRB1*01:01, we created chimeric peptides representing combinations of N- and C-terminal parts of HA_306−318_, CLIP_103−117_, MBP_151−164_, and MBP_88−100_ (Figures 4A,B). Thioredoxin-fused chimeric peptides bearing the C-terminal part from the HA peptide (Trx-[HA], Trx-[MBP_151−156_-HA], Trx-[MBP_88−93_-HA], and Trx-[CLIP-HA]) were loaded on HLA-DRB1*01:01 with or without HLA-DM with a similar rate regardless of the N-terminal part. Conversely, none of the chimeric peptides assembled from the C-terminal part of MBP_151−164_ or MBP_88−100_ peptides (Trx-[MBP_151−164_], Trx-[HA-MBP_157−164_], Trx-[CLIP-MBP_157−164_], Trx-[MBP_88−100_], Trx-[HA-MBP_94−100_]) were capable of efficiently binding HLA-DRB1*01:01. Similar to these findings, substitution of the N-terminal part of the CLIP by fragments from either HA or MBP_151−164_ (Trx-[HA-CLIP]and Trx-[MBP_151−156_-CLIP]) had only a moderate effect on the HLA-DRB1*01:01 binding.

In order to elucidate if there is a similar dependence on the peptide C-terminus in case of HLA-DRB1*15:01 and MBP_85−97_, we created chimeric peptide representing fusion of N-terminal part of pp65_109−123_ [antigenic peptide from cytomegalovirus structural protein (34) and C-terminal part of MBP_85−97_ (Figure 4C). All peptides (Trx-[pp65], Trx-[pp65-MBP_91−97_], and Trx-[MBP_85−97_]) were loaded on HLA-DRB1*15:01 with or without HLA-DM with a similarly high rate.

### C-Terminal Part of Myelin Peptide Significantly Restricts Its Ability to Compete With HA Peptide in Terms of Loading on HLA-DRB1*01:01

**Figure 5:**
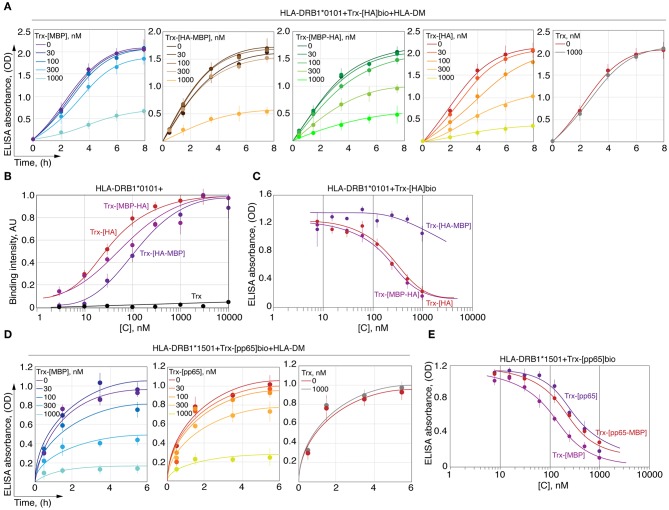
HA peptide prevents loading of MBP_146−170_ onto the HLA-DRB1*01:01. **(A)** Recombinant HLA-DRB1*01:01 (150 nM) and HLA-DM (150 nM) were incubated with biotinylated thioredoxin-fused HA_306−318_ (Trx-[HA]bio) (150 nM) in the presence of increasing concentrations (0-1 μM) of thioredoxin-fused MBP_151−164_ (Trx-[MBP]), HA_306−311_-MBP_157−164_ (Trx-[HA-MBP]), MBP_151−156_-HA_312−318_ (Trx-[MBP-HA]) and HA_306−318_ (Trx-[HA]). Thioredoxin (Trx) without any peptide was used as a control. At indicated timepoints 8, 6, 4, 2, and 0 h, the amount of bound Trx-[HA]bio was determined by addition of streptavidin-HRP. **(B)** Binding of thioredoxin fusions containing HA_306−318_ (red) and chimeric peptides assembled from N- and C-terminal parts of HA_306−318_ and MBP_151−164_ (Trx-[MBP-HA] and Trx-[HA-MBP]) in different concentrations (3 nM−10 μM) with recombinant HLA-DRB1*01:01 (5 nM) measured by DELFIA with time-resolved fluorescence detection. **(C)** Recombinant HLA-DRB1*01:01 (150 nM) was incubated with biotinylated thioredoxin-fused HA_306−318_ (Trx-[HA]bio) (150 nM) in the presence of increasing concentrations (7.8 nM−1 μM) of thioredoxin-fused HA_306−318_ (Trx-[HA]) and chimeric peptides assembled from N- and C-terminal parts of HA_306−318_ and MBP_151−164_ (Trx-[MBP-HA] and Trx-[HA-MBP]). After 18 h of incubation, the amount of bound Trx-[HA]bio was determined by addition of streptavidin-HRP. **(D)** Recombinant HLA-DRB1*15:01 (150 nM) and HLA-DM (150 nM) were incubated with biotinylated thioredoxin-fused pp65_109−123_ (Trx-[pp65]bio) (150 nM) in the presence of increasing concentrations (0-1 μM) of thioredoxin-fused MBP_85−97_ (Trx-[MBP]), pp65_109−114_-MBP_91−97_ (Trx-[pp65-MBP]), and pp65_109−123_ (Trx-[pp65]). Thioredoxin (Trx) without any peptide was used as a control. At indicated timepoints 5.5, 3.5, 1.5, and 0.5 h, the amount of bound Trx-[pp65]bio was determined by addition of streptavidin-HRP. **(E)** Recombinant HLA-DRB1*15:01 (150 nM) was incubated with biotinylated thioredoxin-fused pp65_109−123_ (Trx-[pp65]bio) (150 nM) in the presence of increasing concentrations (7.8 nM−1 μM) of thioredoxin-fused MBP_85−97_ (Trx-[MBP]), pp65_109−114_-MBP_91−97_ (Trx-[pp65-MBP]), and pp65_109−123_ (Trx-[pp65]). After 18 h of incubation, the amount of bound Trx-[pp65]bio was determined by addition of streptavidin-HRP.

We next determined the ability of MBP_151−164_ to compete with HA_306−318_ for HLA-DRB1*01:01 binding. To determine this, HLA-DRB1*01:01 was incubated with HLA-DM and biotinylated thioredoxin-fused HA_306−318_ (Trx-[HA]bio) in the presence of increasing concentrations of either Trx-HA_306−318_ (Trx-[HA]), Trx-MBP_151−164_ (Trx-[MBP_151−164_]) or their chimeric variants (Figure 5A). We determined that Trx-[MBP_151−164_] and Trx-[HA-MBP_157−164_] failed to compete with Trx-[HA]bio up to a concentration of 1 μM, whereas non-biotinylated Trx-[HA] and Trx-[MBP_151−156_-HA] decreased binding of Trx-[HA]bio starting from a concentration of 30 nM. To verify that observed difference is caused by inappropriate P6/P7 and P9 residues in MBP_151−164_ peptide, as was shown in case of kinetic discrimination of myelin and viral peptides, we determined the affinity of two chimeric peptides Trx-[HA-MBP_157−164_] and Trx-[MBP_151−156_-HA] (Figure 5B) in comparison with their ability to compete with Trx-[HA]bio for HLA-DRB1*01:01 binding (Figure 5C). Our data suggest that the comparable thermodynamically inhibition capacity of Trx-[HA-MBP_157−164_] is up to two orders of magnitude lower than Trx-[HA] and Trx-[MBP_151−156_-HA].

We also determined the ability of MBP_85−97_ to compete with pp65_109−123_ for HLA-DRB1*15:01 binding. To determine this, HLA-DRB1*15:01 was incubated with HLA-DM and biotinylated thioredoxin-fused pp65_109−123_ (Trx-[pp65]bio) in the presence of increasing concentrations of either Trx-pp65_109−123_ (Trx-[pp65]) or Trx-MBP_85−97_ (Trx-[MBP_85−97_]) (Figure 5D). We showed that Trx-[MBP_85−97_] is capable to compete with Trx-[pp65]bio, similar to non-biotinylated Trx-[pp65], decreasing binding of Trx-[pp65]bio starting from a concentration of 100 nM. Our data suggest that thermodynamic inhibition capacity of Trx-[MBP_85−97_] in comparison with Trx-[pp65] and Trx-[pp65-MBP_91−97_] is up to half of order of magnitude greater (Figure 5E).

## Discussion

In the present study, we have shown the strong association of HLA-DRB1*15 and *03 alleles with MS risk and the significant protective effect of HLA-DRB1*01 and *11 alleles in ethnic Russian people. The association between HLA-DRB1*15 allele and MS was previously shown based on the analysis of a limited independent cohort of ethnic Russians (35). For HLA-DRB1*15, which is widely known as the strongest genetic risk factor of MS, we observed that the OR value was equal to 2.84, which is similar to the results obtained for the majority of European populations (OR = 3.08) (18). Published data on the association of HLA-DRB1*03, *01, and *11 alleles with MS in different populations are presented in Table S2. Among 15 studies where these three alleles were investigated simultaneously (see references in Table S2), positive association with HLA-DRB1*03 was observed in five studies, negative association with HLA-DRB1*01 in seven and with *11 only in three reports. Results of the meta-analysis derived for Caucasians in 2011 revealed the associations of carriage (phenotype) frequencies of HLA-DRB1*03 and *01, but not for *11 with MS, and OR values for *03 and *01 alleles were close to those observed in our study (19). Therefore, our data suggest that Russians share MS-associated HLA-*DRB1* *03 and *01 alleles with other Caucasians.

The OR values for HLA-DRB1*15, *03, and *01 were markedly higher in people who are homozygous for these allelic variants in comparison with heterozygotes individuals containing the same alleles (see Figures 1B, **D**). These data revealed a dose-depended effect not only for risk alleles HLA-DRB1*15 and *03, which was shown earlier (36), but also for the protective allele HLA-DRB1*01. For all genotypes containing one protective and one risk allele, we observed no significant differences in genotype frequencies between MS patients and healthy controls. The small number of persons carrying each of these heterozygous genotypes among patients or healthy individuals (from 5 to 32 persons) do not allow to reach definitive conclusions but estimated OR values close to 1 as well as relatively narrow CIs suggest the mutual compensation of allelic effects in heterozygotes. The most prominent compensatory effect was observed for the genotype HLA-DRB1*01/*15 [OR = 0.94 (CI: 0.47–1.91)]. Although the exact mechanisms by which HLA products encoded by different *DRB1* alleles contribute to MS susceptibility are still unknown, the parameters of autoantigen binding to HLA proteins may be the key component determining predisposing or protective effects of HLA allelic variants on MS development.

**Figure 6:**
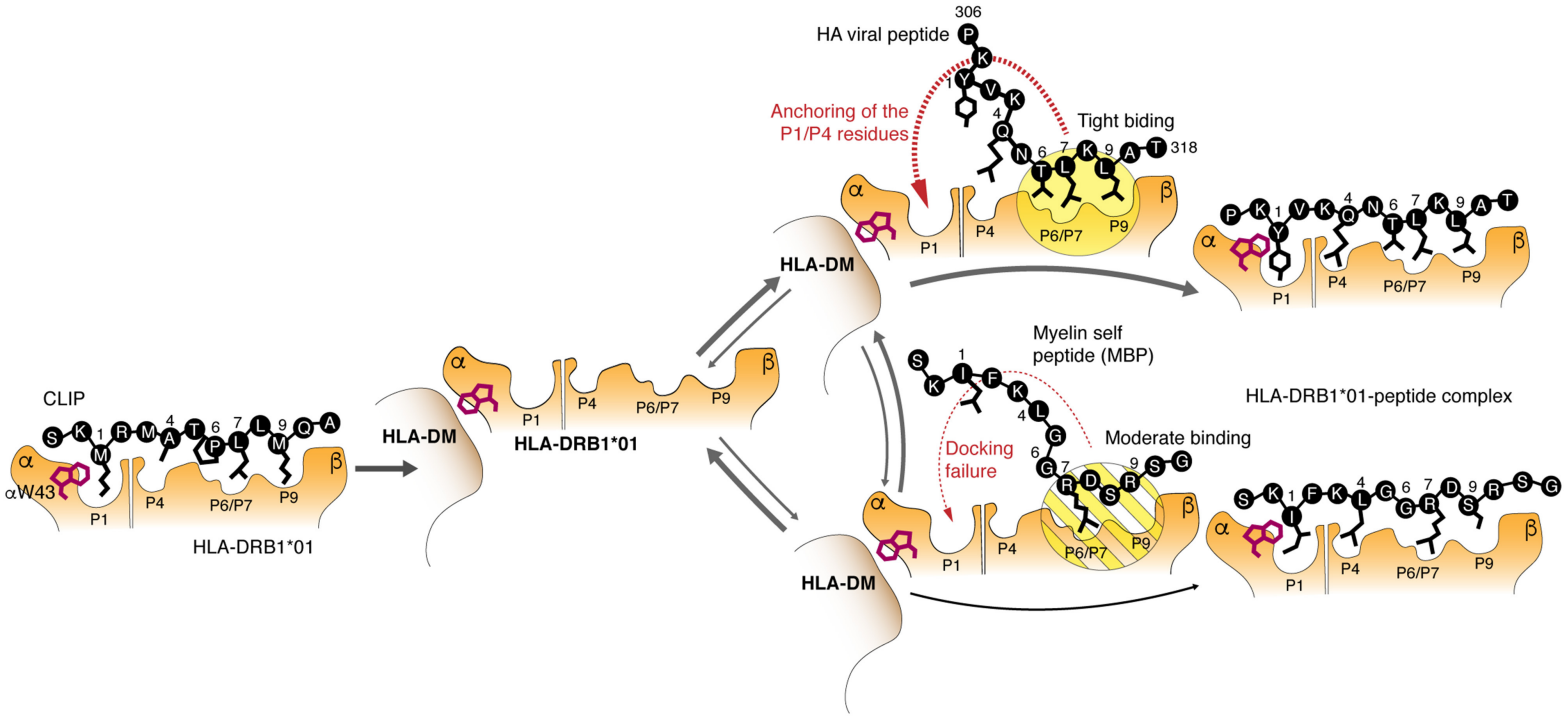
Molecular mechanism of the kinetic selection mediated by the HLA-DM–HLA-DRB1*01:01 complex, which restricts loading of the autoantigenic peptides. Release of the CLIP is followed by assembling of the HLA-DM–HLA-DRB1*01:01 complex, where tryptophan 43 of the HLA α chain is trapped by the HLA-DM. Myelin and exogenous (viral HA as representative example) peptides are competing with each other to bind this bimolecular complex utilizing P6/P7 and P9 residues. Moderate binding of polar P6/P7 and P9 residues in myelin peptides in contrast to the beneficial hydrophobic anchors in the HA peptide does not provide enough time for P1 residue to attack a respective pocket for complete docking.

We have found that the most abundant HLA-DRB1*01:01 allelic variant binds C-terminal and encephalitogenic peptide fragments of MBP with affinity comparable to that of exogenous viral peptides, such as we considered in this work, or even endogenous peptides with high affinity and slow dissociation kinetics (37). Loading of the peptide is mediated by a multistep mechanism including release of the CLIP, which is facilitated by the binding of HLA-DM to HLA-DRB1*01:01; tryptophan 43 of alpha chain of the HLA-DR protein plays an essential role in the binding (12) (Figure 6). Further, P6-P9 pockets are occupied by the C-terminal end of the peptide that facilitates interactions of the N-terminal “head” of the peptide with the P1 and P4 pockets followed by the release of the HLA-DM. These data provide evidence that HLA class II molecules are capable to discriminate kinetically between self- and exogenous peptides during HLA-DM-catalyzed CLIP exchange. Importantly, the affinity of the peptides for the HLA protein are very similar, while the on-rate of their loading onto the HLA are very different. This indicates that the binding kinetics of HLA ligands may be more essential characteristic as opposed to the affinity of the binding and is more relevant physiologically. It should be emphasized that nature proposes at least several other mechanisms to avoid autoimmunity in case of HLAs still capable for self-peptides loading. Molecular dynamics simulation of HLA-DR2—peptide interaction in the absence of DM reveled that protective allele DRB1*16:01 in contrast to the predisposing allele DRB1*15:01 forms more stable complex with the self-peptide in comparison with the viral one. This difference is further reasoned by more tight interaction of the C-terminal part of the self MBP peptide with DRB1*16:01. These data suggest that weak binding of HLA with mimicking viral peptide in case of presence of high affinity self-peptide may serve as a protective factor (38, 39). Recent reports also demonstrate that HLA molecules with high affinity toward self-antigens and associated with autoimmune protection may “steal epitopes” (40) or induce self-epitope specific T regulatory cells (41).

Overall, the data suggest that the binding of MBP_153−161_ and MBP_88−100_ epitopes to HLA-DRB1*01:01 is inefficient, particularly at the first stage, because the P7 pocket is occupied by the basic bulky arginine residue or structurally unfavorable proline, respectively, instead of the highly hydrophobic leucine residue. Therefore, a moderate interaction of the peptide with P6/7 and P9 pockets in contrast to the binding of HA peptide leads to the P1 and P4 docking failure resulting in the peptide dissociation and release of the empty HLA-DM-HLA-DR complex (Figure 6). Because the time for HLA class II loading in the late endosome is restricted to several hours (42), low rate of peptide binding becomes more critical than high affinity of their interactions resulting in the inability to compete with peptides having fast kinetics and even lower affinity. For that reason, the HA peptide completely blocks loading of the myelin peptide onto the HLA-DRB1*01:01 in the equimolar concentration. Thus, it is unlikely that myelin-derived peptides will be presented in the context of the HLA-DRB1*01:01 on the cell surface of the antigen presenting cells at sufficient density required to initiate T cell response. It appears that protective properties of the HLA-DRB1*01 allele may be directly linked to the ability of HLA-DRB1*01:01 to kinetically discriminate between myelin and exogenous peptides. From the opposite side, risk allele HLA-DRB1*15:01 is capable to rapidly present myelin-derived peptides even in competition with exogenous peptides, such as viral pp65_109−123_. *Ex vivo* analysis of HLA-associated peptidome from heterozygous HLA-DRB1*01/*15-positive dendritic cells revealed presence of MBP fragments related to the HLA-DRB1*15 but not to HLA-DRB1*01. Even so the observed compensatory effect of protective HLA-DRB1*01 and MS-predisposing *15 alleles that are co-dominantly expressed in heterozygotes may be explained by dispersion of MBP-loaded HLA-DRB1*15 complexes by HLA-DRB1*01 molecules within MHC clusters, occurring in the immunological synapses formed between T cells and antigen presenting cells (43). Indeed, the density of functional MHC molecules within membrane clusters has proved to be an essential factor regulating T cell responses (44).

MS therapeutic glatiramer acetate (GA) or Copaxone is a 40–100 amino acids polypeptide of a random sequence composed from alanine, lysine, glutamate, and tyrosine at a molar ratio of 4.5:3.6:1.5:1, respectively, that with high affinity binds directly to the purified HLA-DR1, -DR2, and -DR4 molecules (45). Combinatorial chemistry, which lays in the basis of GA, may result in assembling of multiple HLA class II epitopes, that at least in part may be not simply thermodynamically but rather kinetically preferable. Concluding our data provide novel vector of optimization of altered peptide ligands in terms of kinetic discrimination of HLA class II antigens.

Future studies should determine if the proposed molecular mechanism of antigenic peptide loading to MHC II suggesting the kinetic discrimination step may have a more general significance in protecting humans from autoimmunity along with the central tolerance established during negative thymic selection of developing T cells.

## Ethics Statement

This study was carried out in accordance with the recommendations of local ethics committee of the Moscow Multiple Sclerosis Center. All subjects gave written informed consent in accordance with the Declaration of Helsinki.

## Author Contributions

AM, NV, IF, and MZ designed and performed experiments. AM, IK, AF, ISm, and ABe were responsible for statistical analysis and graphic design. AM, OK, OF, AG, and ABe designed the research. IK, VB, and NB performed data collection. MZ, ABo, YS, OF, and AG made intellectual contributions to data analysis, discussion, and coordination of the research team. ABo participated in MS diagnosis and sample collection. RZ performed LC-MS/MS analysis. AP, ISh, and MK performed CFSE proliferation assay. AM, OK, OF, YS, VV, AG, and ABe analyzed data and wrote the manuscript. All authors approved the final manuscript.

### Conflict of Interest

The authors declare that the research was conducted in the absence of any commercial or financial relationships that could be construed as a potential conflict of interest.

## References

[1] Todd JA Acha-Orbea H Bell JI Chao N Fronek Z Jacob CO. A molecular basis for MHC class II–associated autoimmunity. Science. (1988) 240:1003–9. 10.1126/science.3368786 3368786

[2] Nepom GT Erlich H. MHC class-II molecules and autoimmunity. Annu Rev Immunol. (1991) 9:493–525. 10.1146/annurev.iy.09.040191.002425 1910687

[3] Seliger B Kloor M Ferrone S. HLA class II antigen-processing pathway in tumors: molecular defects and clinical relevance. Oncoimmunology. (2017) 6:1–10. 10.1080/2162402X.2016.1171447 28344859 PMC5353941

[4] Prentice HA Tomaras GD Geraghty DE Apps R Fong Y Ehrenberg PK. HLA class II genes modulate vaccine-induced antibody responses to affect HIV-1 acquisition. Sci Transl Med. (2015) 7:296ra112. 10.1126/scitranslmed.aab4005 26180102 PMC4911012

[5] Huppa JB Davis MM. T-cell-antigen recognition and the immunological synapse. Nat Rev Immunol. (2003) 3:973–83. 10.1038/nri1245 14647479

[6] Cresswell P. Assembly, transport, and function of MHC class II molecules. Annu Rev Immunol. (1994) 12:259–93. 10.1146/annurev.iy.12.040194.001355 8011283

[7] Ghosh P Amaya M Mellins E Wiley DC. The structure of an intermediate in class II MHC maturation: CLIP bound to HLA-DR3. Nature. (1995) 378:457–62. 10.1038/378457a0 7477400

[8] Jasanoff a Park SJ Wiley DC. Direct observation of disordered regions in the major histocompatibility complex class II-associated invariant chain. Proc Natl Acad Sci USA. (1995) 92:9900–4. 10.1073/pnas.92.21.9900 7568241 PMC40910

[9] Sherman MA Weber DA Jensen PE. DM enhances peptide binding to class II MHC by release of invariant chain-derived peptide. Immunity. (1995) 3:197–205. 10.1016/1074-7613(95)90089-6 7648393

[10] Weber DA Evavold BD Jensen PE. Enhanced dissociation of HLA-DR-bound peptides in the presence of HLA-DM. Science. (1996) 274:618–20. 10.1126/science.274.5287.618 8849454

[11] Roche PA Furuta K. The ins and outs of MHC class II-mediated antigen processing and presentation. Nat Rev Immunol. (2015) 15:203–16. 10.1038/nri3818 25720354 PMC6314495

[12] Pos W Sethi DK Call MJ Schulze MSED Anders AK Pyrdol J. Crystal structure of the HLA-DM-HLA-DR1 complex defines mechanisms for rapid peptide selection. Cell. (2012) 151:1557–68. 10.1016/j.cell.2012.11.025 23260142 PMC3530167

[13] Li Y Li H Martin R Mariuzza RA. Structural basis for the binding of an immunodominant peptide from myelin basic protein in different registers by two HLA-DR2 proteins. J Mol Biol. (2000) 304:177–88. 10.1006/jmbi.2000.4198 11080454

[14] Smith KJ Pyrdol J Gauthier L Wiley DC Wucherpfennig KW. Crystal Structure of HLA-DR2 (DRA*0101, DRB1*1501) complexed with a peptide from human myelin basic protein. J Exp Med. (1998) 188:1511–20. 10.1084/jem.188.8.1511 9782128 PMC2213406

[15] Yin Y Li Y Mariuzza RA. Structural basis for self-recognition by autoimmune T-cell receptors. Immunol Rev. (2012) 250:32–48. 10.1111/imr.12002 23046121

[16] Hemmer B Kerschensteiner M Korn T. Role of the innate and adaptive immune responses in the course of multiple sclerosis. Lancet Neurol. (2015) 14:406–19. 10.1016/S1474-4422(14)70305-9 25792099

[17] Sawcer S Franklin RJM Ban M. Multiple sclerosis genetics. Lancet Neurol. (2014) 13:700–9. 10.1016/S1474-4422(14)70041-9 24852507

[18] Canto E Oksenberg JR. Multiple sclerosis genetics. Mult Scler. (2018) 24:75–9. 10.1177/1352458517737371 29307290

[19] Zhang Q Lin CY Dong Q Wang J Wang W. Relationship between HLA-DRB1 polymorphism and susceptibility or resistance to multiple sclerosis in Caucasians: a meta-analysis of non-family-based studies. Autoimmun Rev. (2011) 10:474–81. 10.1016/j.autrev.2011.03.003 21440682

[20] Ramagopalan SV Ebers GC. Multiple sclerosis: major histocompatibility complexity and antigen presentation. Genome Med. (2009) 1:1–5. 10.1186/gm105 19895714 PMC2808740

[21] Yin Y Li Y Kerzic MC Martin R Mariuzza RA. Structure of a TCR with high affinity for self-antigen reveals basis for escape from negative selection. EMBO J. (2011) 30:1137–48. 10.1038/emboj.2011.21 21297580 PMC3061028

[22] Hahn M Nicholson MJ Pyrdol J Wucherpfennig KW. Unconventional topology of self peptide-major histocompatibility complex binding by a human autoimmune T cell receptor. Nat Immunol. (2005) 6:490–6. 10.1038/ni1187 15821740 PMC3415330

[23] Li Y Huang Y Lue J Quandt JA Martin R Mariuzza RA. Structure of a human autoimmune TCR bound to a myelin basic protein self-peptide and a multiple sclerosis-associated MHC class II molecule. EMBO J. (2005) 24:2968–79. 10.1038/sj.emboj.7600771 16079912 PMC1201352

[24] Polman CH Reingold SC Banwell B Clanet M Cohen JA Filippi M. Diagnostic criteria for multiple sclerosis: 2010 revisions to the McDonald criteria. Ann Neurol. (2011) 69:292–302. 10.1002/ana.22366 21387374 PMC3084507

[25] Sreevalsan T. Isolation of dendritic cells from human blood for *in vitro* interaction studies with fungal antigens. Methods Mol Biol. (2009) 499:1–8. 10.1007/978-1-60327-151-6_1 19152033

[26] Markov OV Mironova NL Shmendel EV Serikov RN Morozova NG Maslov MA. Multicomponent mannose-containing liposomes efficiently deliver RNA in murine immature dendritic cells and provide productive anti-tumour response in murine melanoma model. J Control Release. (2015) 213:45–56. 10.1016/j.jconrel.2015.06.028 26134071

[27] Rappsilber J Mann M Ishihama Y. Protocol for micro-purification, enrichment, pre-fractionation and storage of peptides for proteomics using StageTips. Nat Protoc. (2007) 2:1896–906. 10.1038/nprot.2007.261 17703201

[28] Ma B Zhang K Hendrie C Liang C Li M Doherty-Kirby A Lajoie G. PEAKS: Powerful software for peptide *de novo* sequencing by tandem mass spectrometry. Rapid Commun Mass Spectrom. (2003) 17:2337–42. 10.1002/rcm.1196 14558135

[29] Mamedov AE Ponomarenko NA Belogurov AA Gabibov AG. Erratum to: heterodimer HLA-DM fused with constant fragment of the heavy chain of the human immunoglobulin accelerates influenza hemagglutinin peptide HA306-318 loading to HLA-DR1. Bull Exp Biol Med. (2016) 161:442–6. 10.1007/s10517-016-3353-y 27520960

[30] Belogurov AA Kurkova IN Friboulet A Thomas D Misikov VK Zakharova MY. Recognition and degradation of myelin basic protein peptides by serum autoantibodies: novel biomarker for multiple sclerosis. J Immunol. (2008) 180:1258–67. 10.4049/jimmunol.180.2.1258 18178866

[31] Stern LJ Brown JH Jardetzky TS Gorga JC Urban RG Strominger JL. Crystal structure of the human class II MHC protein HLA-DR1 complexed with an influenza virus peptide. Nature. (1994) 368:215–21. 10.1038/368215a0 8145819

[32] Anders AK Call MJ Schulze MS Fowler KD Schubert DA Seth NP. HLA-DM captures partially empty HLA-DR molecules for catalyzed removal of peptide. Nat Immunol. (2011) 12:54–61. 10.1038/ni.1967 21131964 PMC3018327

[33] Yin L Maben ZJ Becerra A Stern LJ. Evaluating the role of HLA-DM in MHC Class II–peptide association reactions. J Immunol. (2015) 195:706–16. 10.4049/jimmunol.1403190 26062997 PMC4490944

[34] Muixi L Carrascal M Alvarez I Daura X Marti M Armengol MP. Thyroglobulin peptides associate *in vivo* to HLA-DR in autoimmune thyroid glands. J Immunol. (2008) 181:795–807. 10.4049/jimmunol.181.1.795 18566446

[35] Sudomoina MA Boiko AN Demina TL Gusev EI Boldyreva MN Trofimov DY Association of multiple sclerosis in the Russian population with HLA-DRB1 gene alleles. Mol Biol. (1998) 32:255–60. 9608945

[36] Barcellos LF Sawcer S Ramsay PP Baranzini SE Thomson G Briggs F. Heterogeneity at the HLA-DRB1 locus and risk for multiple sclerosis. Hum Mol Genet. (2006) 15:2813–24. 10.1093/hmg/ddl223 16905561

[37] Yin L Trenh P Guce A Wieczorek M Lange S Sticht J. Susceptibility to HLA-DM protein is determined by a dynamic conformation of major histocompatibility complex class II molecule bound with peptide. J Biol Chem. (2014) 289:23449–64. 10.1074/jbc.M114.585539 25002586 PMC4156084

[38] Kumar A Cocco E Atzori L Marrosu MG Pieroni E. Structural and dynamical insights on HLA-DR2 complexes that confer susceptibility to multiple sclerosis in sardinia: a molecular dynamics simulation study. PLoS ONE. (2013) 8:e59711. 10.1371/journal.pone.0059711 23555757 PMC3608583

[39] Kumar A Melis P Genna V Cocco E Marrosu MG Pieroni E. Antigenic peptide molecular recognition by the DRB1-DQB1 haplotype modulates multiple sclerosis susceptibility. Mol Biosyst. (2014) 10:2043–54. 10.1039/C4MB00203B 24853027

[40] van Lummel M Buis DTP Ringeling C de Ru AH Pool J Papadopoulos GK. Epitope stealing as a mechanism of dominant protection by HLA-DQ6 in type 1 diabetes. Diabetes. (2019) 68:787–95 10.2337/db18-0501 30626607

[41] Ooi JD Petersen J Tan YH Huynh M Willett ZJ Ramarathinam SH. Dominant protection from HLA-linked autoimmunity by antigen-specific regulatory T cells. Nature. (2017) 545:243–7. 10.1038/nature22329 28467828 PMC5903850

[42] Rabinowitz JD Vrljic M Kasson PM Liang MN Busch R Boniface JJ. Formation of a highly peptide-receptive state of class II MHC. Immunity. (1998) 9:699–709. 10.1016/S1074-7613(00)80667-6 9846491

[43] Vogt AB Spindeldreher S Kropshofer H. Clustering of MHC-peptide complexes prior to their engagement in the immunological synapse: lipid raft and tetraspan microdomains. Immunol Rev. (2002) 189:136–51. 10.1034/j.1600-065X.2002.18912.x 12445271

[44] Anikeeva N Gakamsky D Schøller J Sykulev Y. Evidence that the density of self peptide-MHC ligands regulates T-cell receptor signaling. PLoS ONE. (2012) 7:e41466. 10.1371/journal.pone.0041466 22870225 PMC3411518

[45] Fridkis-Hareli M Strominger JL. Promiscuous binding of synthetic copolymer 1 to purified HLA-DR molecules. J Immunol. (1998) 160:4386–97. 9574543

