# Supplementary material for: Protective Allele for Multiple Sclerosis HLA-DRB1*01:01 Provides Kinetic Discrimination of Myelin and Exogenous Antigenic Peptides
Source: Front Immunol. 2020 Jan 17;10:3088. doi: 10.3389/fimmu.2019.03088 (PMC6978714; doi:10.3389/fimmu.2019.03088)
Supplement: Supplementary file 1 [file Presentation_1.PDF]

Supplemental information

Protective allele for multiple sclerosis HLA-DRB1*0101 provides kinetic discrimination of myelin and exogenous antigenic peptides

by Mamedov et al.

Supplemental Information includes Tables S1-S2, Figures S1-S2, Supplemental Methods and Supplementary References 1-15

Table S1. Association of HLA-*DRB1* alleles carriage with MS.

HLA-*DRB1*, groups of alleles	Carriers, n (%)		p_f values	p_{perm} values	OR (95% CI)
	MS cases, N=565	Healthy individuals, N=471			
*DRB1**01	95 (16.8)	127 (26.9)	**$5.16{\times}10^{-5}$**	**0.00062**	**0.55 (0.41-0.74)**
*DRB1**03	118 (20,9)	61 (13,0)	**0.00047**	**0.0056**	**1.77 (1.27-2.49)**
*DRB1**04	107 (18,9)	103 (22,3)	0.138	1.00	0.83 (0.61-1.13)
*DRB1**07	111 (19,6)	116 (24,6)	**0.032**	0.38	0.75 (0.56-1.01)
*DRB1**08	48 (8,5)	40 (8,5)	0.55	1.00	1.00 (0.65-1.55)
*DRB1**09	4 (0,7)	14 (3,0)	**0.0052**	0.062	**0.23 (0.077-0.71)**
*DRB1**10	11 (1,9)	11 (2,3)	0.83	1.00	0.83 (0.36-1.93)
*DRB1**11	99 (17,5)	129 (27,4)	**$9.35{\times}10^{-5}$**	**0.0011**	**0.56 (0.42-0.76)**
*DRB1**12	21 (3,7)	36 (7,6)	**0.0044**	0.053	**0.47 (0.27-0.81)**
*DRB1**13	141 (25,0)	102 (21,7)	0.12	1.00	1.20 (0.90-1.60)
*DRB1**14	6 (1,1)	9 (1,9)	0.19	1.00	0.55 (0.19-1.56)
*DRB1**15	264 (46,7)	111 (23,6)	**$4.86{\times}10^{-15}$**	**$5.8{\times}10^{-14}$**	**2.84 (2.17-3.72)**
*DRB1**16	30 (5,3)	29 (6,2)	0.33	1.00	0.85 (0.50-1.45)

MS – multiple sclerosis; OR – odds ratio; CI – confidential interval.
Fisher *p*-values (p_f), permutation *p*-values (p_{perm}) and OR (95% CI) values are represented in the table.
Significant values are shown in bold.

Table S2. Association of HLA-*DRB1**01, *03 and *11 alleles with MS risk in different populations.

Ethnicity	Sample size	Published data	Reference
Colombian	103 MS *vs* 202 HI	*DRB1**01 – no association<br>*DRB1**03 – no association<br>*DRB1**11 – no association	(1)
Slovak	282 MS *vs* 238 HI	*DRB1**01 – no association<br>*DRB1**03 – no association<br>*DRB1**11 – no association	(2)
Iranian	73 MS *vs* 40 HI	*DRB1**01 – no association<br>*DRB1**03 – positive association with MS (OR = 5.66, p = 0.0021)<br>*DRB1**11 – no association	(3)
Sardinian	2555 MS *vs* 1365 HI	*DRB1**01 – no association<br>*DRB1**0301 – positive association with MS in the haplotype with D*QB1**02:01 (OR = 1.7, p_c = 7.9×10^{-22})<br>*DRB1**11 – negative association with MS (OR = 0.8, p_c = 0.027)	(4)
African Americans	1162 MS *vs* 2092 HI	*DRB1**0101 – no association<br>*DRB1**0301 – positive association with MS (OR = 1.54, p = 3.01×10^{-5})<br>*DRB1**1101 – negative association with MS (OR = 0.61, p = 0.002)	(5)
Swedish and Norwegian	1784 MS *vs* 1660 HI	*DRB1**01 – negative association with MS (OR = 0.82, p=0.034).<br>*DRB1**03 – no association<br>*DRB1**11 – no association	(6)
Lithuanian	120 MS *vs* 120 HI	*DRB1**01 – negative association with MS (OR = 0.56, p < 0.0001)<br>*DRB1**03 – negative association with MS (OR = 0.69, p = 0.034)<br>*DRB1**11 – no association	(7)
Japanese	108 MS *vs* 127 HI	*DRB1**01 – negative association with MS (OR = 0.394, p_c = 0.0406)<br>*DRB1**03 – no association<br>*DRB1**11 – no association	(8)
Brazilian	119 MS *vs* 305 HI	*DRB1**01 – no association<br>*DRB1**03 – no association<br>*DRB1**11 – no association	(9)
Basque	197 MS *vs* 200 HI	*DRB1**0101 – negative association with MS (OR = 0.284, p = 0.00003)<br>*DRB1**0301 – no association<br>*DRB1**1101 – no association	(10)
Spanish	21 MS *vs* 156 HI	*DRB1**01 – negative association with MS (14% vs 31%, p_c = 0.011)<br>*DRB1**03 – no association<br>*DRB1**11 – no association	(11)
Canadian	TDT-test,<br>1432 MS families	*DRB1**01 – the resistance allele in HLA-*DRB1**01/15 carriers<br>*DRB1**03 – no association<br>*DRB1**11 – the resistance allele	(12)
Caucasians from USA and UK	PDT-test,<br>1019 MS families	*DRB1**01 – no association<br>*DRB1**03 – the risk allele in the homozygous genotype HLA-*DRB1**03/03<br>*DRB1**11 – no association	(13)
Finnish	TDT-test,<br>249 MS families	*DRB1**01 –the resistance allele in haplotype HLA-*DRB1**01-*DQB1**0501<br>*DRB1**03 – no association<br>*DRB1**11– no association	(14)
Sardinian	240 MS *vs* 170 HI	*DRB1**01 – no association<br>*DRB1**0301 – positive association with MS (31% vs 21%, p_c = 0.003)<br>*DRB1**11 – no association	(15)

HI – healthy individuals; MS – multiple sclerosis; OR – odds ratio.

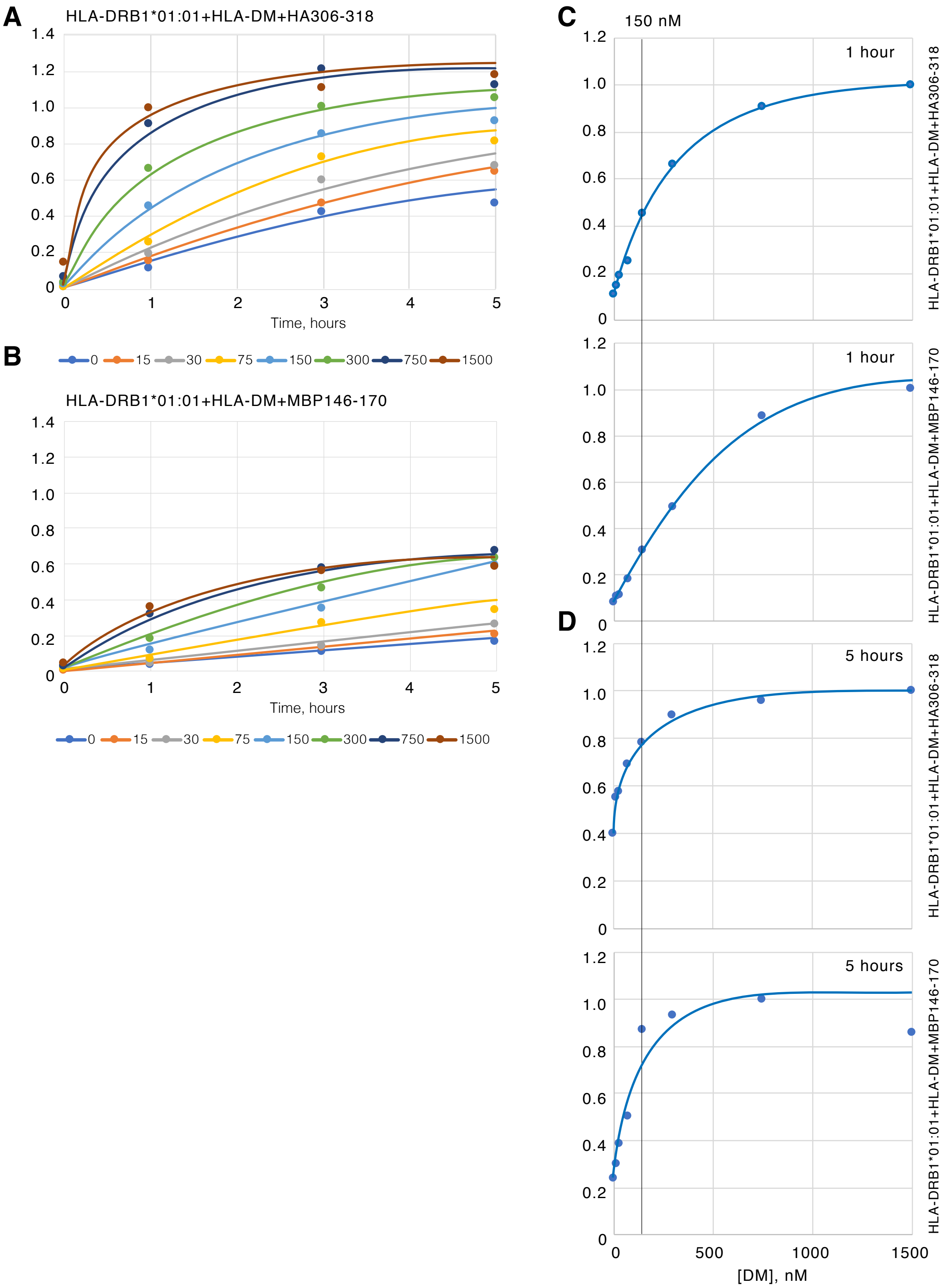

Figure S1. Kinetics of binding of biotinylated thioredoxin-fused peptides HA306-318 **(A)** and MBP146-170 **(B)** (150 nM) with HLA-DRB1*01:01 (150 nM), catalyzed by HLA-DM in the range of increasing concentrations (15 nM – 1.5 μM). HLA-DM concentration dependences are presented for 1h **(C)** and 5h **(D)** of incubation.

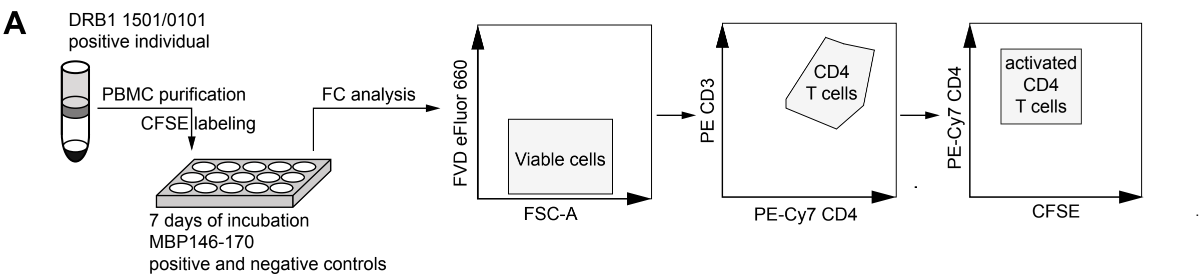

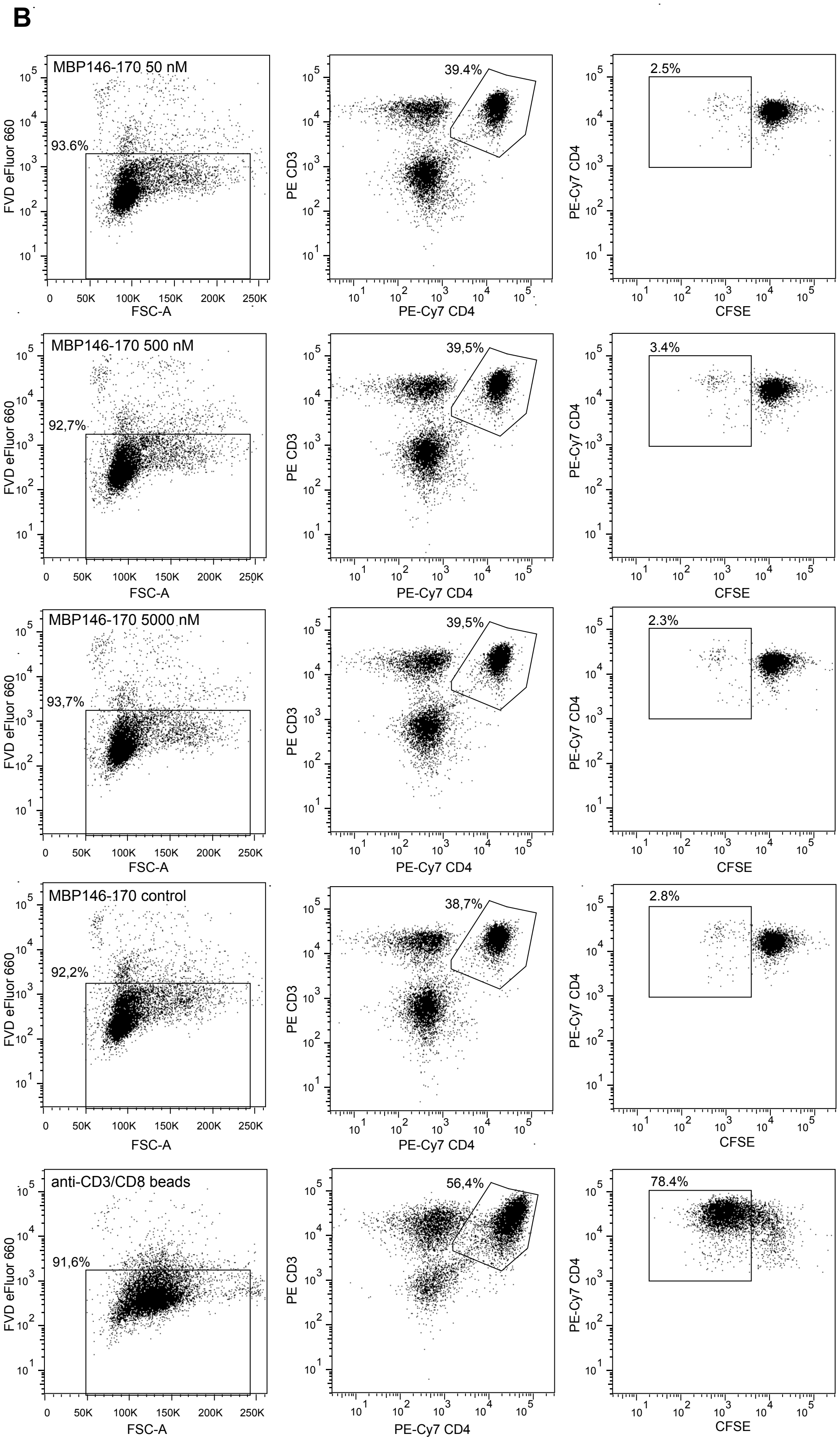

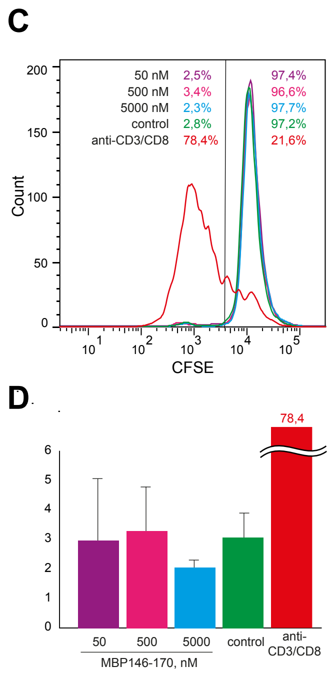

Figure S2. CFSE prolifiration assay. (A) Human PBMC isolated from individual with heterozygous genotype HLA-*DRB1**01:01/*15:01 were labeled with CFSE and further incubated with $MBP_{146\text{-}170}$ in different concentrations (0-5000 nM) or anti-CD3/CD8 magnetic beads during 7 days. Next, the cells were stained with FVD and anti-CD3 -CD4 antibodies and further analyzed by flow cytometry. (B) From top to bottom: CD4-positive T cells stimulated with $MBP_{146\text{-}170}$ at 50, 500 and 5000 nM, unstimulated cells and cells stimulated with anti-CD3/CD8 magnetic beads. (C) Histogram representing intensity of CFSE fluorescence in variously stimulated CD4-positive T cells analyzed by flow cytometry. (D) Plot demonstrates percentage of proliferating CD4-positive T cells depending on stimulation. Bars represent standard deviation (n=3).

Supplemental methods

CFSE proliferation assay

PBMC were isolated by standard protocol from 30 mL of human peripheral blood. Approximately 30x10^6 PBMCs were collected. For CFSE labelling the cells were washed and dissolved in 1 µM CFSE solution in PBS and further incubated for 20 min at 37°C. Next, cells were washed with PBS supplemented with 5% FBS. The cell pellet was dissolved in UltraCULTURE medium (LONZA) and counted. The cells were further placed in 96-well plates with round bottom (200.000 cells per well) in concentration of 10^6 cells/mL. Proliferation of CD4-positive T cells was analyzed in triplicates. Three different concentrations of $MBP_{146-170}$ (5 µM, 0.5 µM and 0.05 µM) were used. The cells without stimulation and cells stimulated with anti-CD3/CD8 magnetic beads were used as a negative and positive controls, respectively. PBMCs were totally cultivated for 7 days at 37°C and 5% CO_2. Next, the cells were washed with PBS and resuspended in 50 µl of FVD solution (1:1000 dilution, ThermoFisher Scientific) and incubated for 30 min at 4°C in darkness. Further cells were washed with FC buffer (PBS supplemented with 2% FBS and NaN_3), dissolved in 50 µl of 10% Normal Mouse Serum (Invitrogen) in PBS and incubated for 20 min at 4°C in darkness for Fc receptors blocking. After washing the cells were dissolved in 30 µl per well of antibody mix solution in PBS (anti-CD3 PE-conjugated antibody 12-0037-41, ThermoFisher Scientific; anti-CD4 PE-Cy7-conjugated antibody 25-0049-42, ThermoFisher Scientific), 0.5 µg/mL each. Plate was further incubated for 20 min at 4°C in darkness. Next, the cells were washed by FC buffer and resuspended in 300 µl of FC buffer and further analyzed on BD flow cytometer.

Supplementary References

1. Toro J, Cuellar-Giraldo D, Díaz-Cruz C, Burbano LE, Guío CM, Reyes S, Cortes F, Cárdenas-Robledo S, Narváez DM, Cárdenas W, et al. HLA-DRB1* 14 is a protective allele for multiple sclerosis in an admixed colombian population. *Neurol Neuroimmunol NeuroInflammation* (2016) **3**: doi:10.1212/NXI.0000000000000192

2. Michalik J, Čierny D, Kantorová E, Kantárová D, Juraj J, Párnická Z, Kurča E, Dobrota D, Lehotský J. The association of HLA-DRB1 and HLA-DQB1 alleles with genetic susceptibility to multiple sclerosis in the Slovak population. *Neurol Res* (2015) **37**:1060–1067. doi:10.1080/01616412.2015.1115212

3. Abolfazli R, Samadzadeh S, Sabokbar T, Siroos B, Armaki SA, Aslanbeiki B, Ghelman M, Taheri T, Shakoori A. Relationship between HLA-DRB1* 11/15 genotype and susceptibility to multiple sclerosis in IRAN. *J Neurol Sci* (2014) **345**:92–96. doi:10.1016/j.jns.2014.07.013

4. Cocco E, Murru R, Costa G, Kumar A, Pieroni E, Melis C, Barberini L, Sardu C, Lorefice L, Fenu G, et al. Interaction between HLA-DRB1-DQB1 Haplotypes in Sardinian Multiple Sclerosis Population. *PLoS One* (2013) **8**:1–12. doi:10.1371/journal.pone.0059790

5. Isobe N, Gourraud P-A, Harbo HF, Caillier SJ, Santaniello A, Khankhanian P, Maiers M, Spellman S, Cereb N, Yang S, et al. Genetic risk variants in African Americans with multiple sclerosis. *Neurology* (2013) **81**:219–27. doi:10.1212/WNL.0b013e31829bfe2f

6. Link J, Kockum I, Lorentzen AR, Lie B a, Celius EG, Westerlind H, Schaffer M, Alfredsson L, Olsson T, Brynedal B, et al. Importance of human leukocyte antigen (HLA) class I and II alleles on the risk of multiple sclerosis. *PLoS One* (2012) **7**:e36779. doi:10.1371/journal.pone.0036779

7. Balnyte R, Rastenyte D, Mickevičiene D, Vaitkus A, Skrodeniene E, Vitkauskiene A. Frequency of HLA-DRB1 gene alleles in patients with multiple sclerosis in a Lithuanian population. *Med* (2012) **48**:9–14.

8. Isobe N, Matsushita T, Yamasaki R, Ramagopalan S V., Kawano Y, Nishimura Y, Ebers GC, Kira J. Influence of HLA-DRB1 alleles on the susceptibility and resistance to multiple sclerosis in Japanese patients with respect to anti-aquaporin 4 antibody status. *Mult Scler* (2010) **16**:147–155. doi:10.1177/1352458509355067

9. Kaimen-Maciel DR, Vissoci Reiche EM, Borelli SD, Morimoto HK, Melo FC, Lopes J, Dorigon RF, Cavalet C, Yamaguchi EM, Silveira TL, et al. HLA-DRB1* allele-associated genetic susceptibility and protection against multiple sclerosis in Brazilian patients. *Mol Med Rep* (2009) **2**:993–998. doi:10.3892/mmr-00000204

10. Fernández O, R-Antigüedad A, Pinto-Medel MJ, Mendibe MM, Acosta N, Oliver B, Guerrero M, Papais-Alvarenga M, Fernández-Sánchez V, Leyva L. HLA class II alleles in patients with multiple sclerosis in the Biscay province (Basque Country, Spain). *J Neurol* (2009) **256**:1977–1988. doi:10.1007/s00415-009-5223-2

11. Fernandez-Morera JL, Rodriguez-Rodero S, Tunon A, Martinez-Borra J, Vidal-Castineira JR, Lopez-Vazquez A, Rodrigo L, Rodrigo P, González S, Lahoz CH, et al. Genetic influence of the nonclassical major histocompatibility complex class I molecule MICB in multiple sclerosis susceptibility. *Tissue Antigens* (2008) **72**:54–59. doi:10.1111/j.1399-0039.2008.01066.x

12. Ramagopalan S V., Morris AP, Dyment DA, Herrera BM, DeLuca GC, Lincoln MR, Orton SM, Chao MJ, Sadovnick AD, Ebers GC. The inheritance of resistance alleles in multiple sclerosis. *PLoS Genet* (2007) **3**:1607–1613. doi:10.1371/journal.pgen.0030150

13. Barcellos LF, Sawcer S, Ramsay PP, Baranzini SE, Thomson G, Briggs F, Cree BCA, Begovich AB, Villoslada P, Montalban X, et al. Heterogeneity at the HLA-DRB1 locus and risk for multiple sclerosis. *Hum Mol Genet* (2006) **15**:2813–2824. doi:10.1093/hmg/ddl223

14. Laaksonen M, Pastinen T, Sjöroos M, Kuokkanen S, Ruutiainen J, Sumelahti ML, Reijonen H, Salonen R, Wikström J, Panelius M, et al. HLA class II associated risk and protection against multiple sclerosis-a Finnish family study. *J Neuroimmunol* (2002) **122**:140–5. Available at: http://www.ncbi.nlm.nih.gov/pubmed/11777553

15. Marrosu MG, Murru MR, Costa G, Cucca F, Sotgiu S, Rosati G, Muntoni F. Multiple sclerosis in Sardinia is associated and in linkage disequilibrium with HLA-DR3 and -DR4 alleles. *Am J Hum Genet* (1997) **61**:454–7. doi:S0002-9297(07)64074-9 [pii]\r10.1016/S0002-9297(07)64074-9 [doi]
